# Pathways of Pathogenicity: Transcriptional Stages of Germination in the Fatal Fungal Pathogen *Rhizopus delemar*

**DOI:** 10.1128/mSphere.00403-18

**Published:** 2018-09-26

**Authors:** Poppy C. S. Sephton-Clark, Jose F. Muñoz, Elizabeth R. Ballou, Christina A. Cuomo, Kerstin Voelz

**Affiliations:** a Institute for Microbiology and Infection, School of Biosciences, University of Birmingham, Birmingham, United Kingdom; b Infectious Disease and Microbiome Program, Broad Institute of MIT and Harvard, Cambridge, Massachusetts, USA; Carnegie Mellon University

**Keywords:** RNA-Seq, *Rhizopus delemar*, fungi, germination, mucormycosis, pathogens, spores, time course, transcription

## Abstract

Rhizopus delemar is an invasive fungal pathogen responsible for the frequently fatal disease mucormycosis. Germination, a crucial mechanism by which infectious spores of Rhizopus delemar cause disease, is a key developmental process that transforms the dormant spore state into a vegetative one. The molecular mechanisms that underpin this transformation may be key to controlling mucormycosis; however, the regulation of germination remains poorly understood. This study describes the phenotypic and transcriptional changes that take place over the course of germination. This process is characterized by four distinct stages: dormancy, isotropic swelling, germ tube emergence, and hyphal growth. Dormant spores are shown to be transcriptionally unique, expressing a subset of transcripts absent in later developmental stages. A large shift in the expression profile is prompted by the initiation of germination, with genes involved in respiration, chitin, cytoskeleton, and actin regulation appearing to be important for this transition. A period of transcriptional consistency can be seen throughout isotropic swelling, before the transcriptional landscape shifts again at the onset of hyphal growth. This study provides a greater understanding of the regulation of germination and highlights processes involved in transforming Rhizopus delemar from a single-cellular to multicellular organism.

**IMPORTANCE** Germination is key to the growth of many organisms, including fungal spores. Mucormycete spores exist abundantly within the environment and germinate to form hyphae. These spores are capable of infecting immunocompromised individuals, causing the disease mucormycosis. Germination from spore to hyphae within patients leads to angioinvasion, tissue necrosis, and often fatal infections. This study advances our understanding of how spore germination occurs in the mucormycetes, identifying processes we may be able to inhibit to help prevent or treat mucormycosis.

## Abstract (precis)

Germination is key to the growth of many organisms, including fungal spores. Mucormycete spores exist abundantly within the environment and germinate to form hyphae. These spores are capable of infecting immunocompromised individuals, causing the disease mucormycosis. Germination from spore to hyphae within patients leads to angioinvasion, tissue necrosis, and often fatal infections. This study advances our understanding of how spore germination occurs in the mucormycetes, identifying processes we may be able to inhibit to help prevent or treat mucormycosis.

## INTRODUCTION

Fungal spores are found ubiquitously within the environment and are key to the dispersal and survival of many fungal species (1, 2). Spores can endure severe temperatures, desiccation, and high levels of radiation and radical exposure, conditions fatal to many other life-forms (3). The ability to survive in harsh environments has enabled the spread of fungal spores by wind, water, and animal dispersal across the globe. Once distributed, spores may stay dormant for thousands of years (4), before germination is initiated under favorable conditions.

Germination cues can include, but are not limited to, the introduction of nutrients, the presence of light, temperature modulation, changes in osmolarity, pH shifts, the removal of dormancy factors, and the introduction of extracellular signaling molecules (5–15). Once germination is initiated, spores begin to swell and take up water. At a critical point, the cell polarizes (16) and hyphae emerge from the swollen spore bodies. Given the correct conditions, the transition from dormancy to vegetative hyphal growth can occur in as little as 6 h, allowing the fungi to rapidly colonize favorable environments. Fungal spores are the infectious agents of many fungal diseases (17–19) (e.g., mucormycosis, aspergillosis, blastomycosis, cryptococcosis, coccidioidomycosis, and histoplasmosis). The transition from dormancy to vegetative growth allows for the onset of disease within a host, yet we currently have a limited understanding of the molecular pathways regulating this fundamental developmental process in human-pathogenic fungi (20–28).

Mucormycosis is an emerging fungal infectious disease with an extremely high mortality rate of over 90% in disseminated cases (29). Current antifungal treatments are ineffective, resulting in the reliance upon surgical debridement of infected tissues (30), often leading to long-term disability. Disease can be caused by several species of the Mucorales order; however, Rhizopus delemar, previously known as Rhizopus oryzae, accounts for 70% of cases (31). Spores are the infectious agents of mucormycosis. While immunocompetent individuals control spore germination through phagocytic uptake, mucormycete spores can survive within immune effector cells, causing latent infection (32). In immunocompromised patients, inhibition of spore germination by phagocytes fails, enabling fungal growth (33). Hyphal extension within tissue leads to angioinvasion, thrombosis, tissue necrosis, and eventually death (30, 34). Given the significance of spore germination in mucormycosis pathogenesis, medical interventions that target and inhibit this developmental process might improve patient prognosis. Therefore, we aimed to comprehensively characterize the transcriptional and phenotypic changes that occur over time during this process.

Phenotypic and transcriptional approaches were taken to follow the germination of Rhizopus delemar over time. With the previously annotated genome of Rhizopus delemar (35), shown to have undergone whole-genome duplication, our transcriptome sequencing (RNA-Seq) data were analyzed and used to create an updated gene set. Our data reveal a clear progression of transcriptional regulation over time, linked to observed phenotypic changes. Together, this work represents the most comprehensive analysis of the transcriptional landscape during germination in a human fungal pathogen to date.

## RESULTS

### Phenotypic characteristics of germinating *R. delemar*.

**FIG 1:**
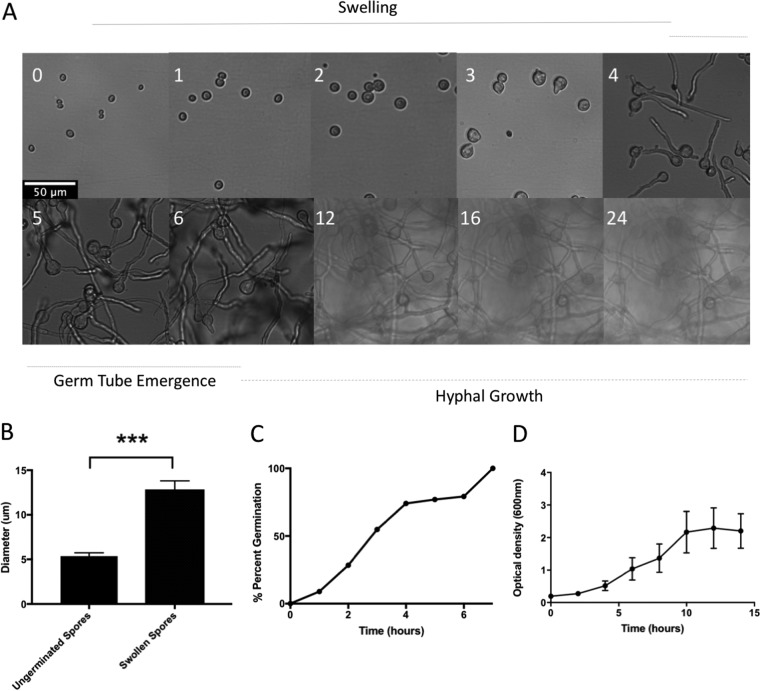
Phenotypic characterization of germinating spores. (A) Spores germinated in SAB were imaged at hours (indicated by white numbers) postgermination. Scale bar = 50 µm for all images. Micrographs representative of >3 replicate experiments are shown. (B) Diameter of ungerminated spore bodies (*n* = 3; time [*T*] = 0 h) compared to spore body size measured immediately prior to germ tube emergence for each spore (*n* = 3; *T* = 4 to 6 h). (C) Spore germination as a percentage over time, determined by live-cell imaging (*n* = 3). (D) Fungal mass over time, determined by optical density at 600 nm.

Germination is characterized by three distinct transitions: dormancy to swelling, swelling to germ tube emergence, and the switch to sustained filamentous growth. This process is common to many filamentous fungi, although the timing of germination varies among species (36). We therefore characterized the phenotypic progression of Rhizopus delemar strain RA 99-880 through germination by live-cell imaging (Fig. 1). The switch from dormancy to swelling was triggered by exposure to rich medium. Swelling, characterized by an isotropic increase in size, continued for 4 to 6 h (Fig. 1A). Between resting and fully swollen, the average spore diameter increased from 5 μm to 13 μm (Fig. 1B). Once fully swollen, germ tubes emerged from the spore bodies. Most spore bodies (75.5% [Fig. 1C]) produced hyphae that exceeded the diameter of the spore body in length by 4 to 5 h. At this time point, the spores were considered fully germinated. Hyphal growth continued from 6 to 24 h, demonstrated by increase in optical density (Fig. 1D), with the average width of hyphae being 5 ± 1.03 μm and the average length being 135 ± 30 μm.

### Transcription over time: experimental design.

Our phenotypic analysis of spore germination established the temporal pattern for the development of spores from dormancy to filamentous growth. These dramatic morphological changes require vast cellular reprogramming. In this study, we performed transcriptional analysis of each stage outlined in this process. For high-resolution capture of the transcriptional regulation of spore germination, we isolated and sequenced mRNA from resting spores (0 h) and swelling spores (1, 2, 3, 4, and 5 h) and during filamentous growth (6, 12, 16, and 24 h). Three biological replicates were produced for each time point, and mRNA from each sample was sequenced with Illumina HiSeq technology, with 100-bp paired end reads. Reads were aligned to the R. delemar genome (35), giving an average alignment rate of over 95% per sample, with an average of 68% (12,170 genes) of all genes expressed over all time points. We utilized our RNA-Seq data to revise the current annotation of the available R. delemar genome, using BRAKER 2.1.0 (37) to improve gene structures and incorporate these into an updated annotation. Compared to the previous annotation (35), this updated set included 475 new predicted genes, 370 new protein family domains (Pfam terms), 103 new pathway predictions (KEGG-EC), and 96 new transmembrane domains (TMHMM terms). The updated annotation was assessed for completeness with BUSCO v3 (38) and was shown to include a good representation of expected core eukaryotic genes, with minimal missing BUSCOs (2%) (see Fig. S1 in the supplemental material).

10.1128/mSphere.00403-18.1 FIG S1 Table displaying genome annotation statistics, comparing the original R. delemar annotation (column 1) and the annotation updated with our RNA-Seq data (column 2). Download FIG S1, TIF file, 0.1 MB. Copyright © 2018 Sephton-Clark et al. 2018 Sephton-Clark et al. This content is distributed under the terms of the Creative Commons Attribution 4.0 International license.

**FIG 2:**
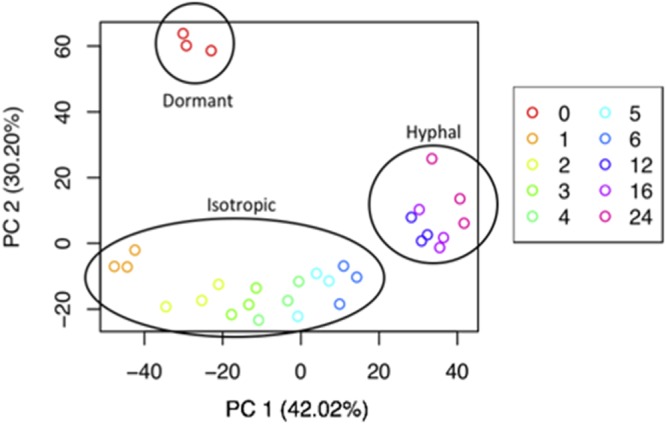
Principal-component analysis of 7,942 genes differentially expressed across all time points (*n* = 3 for each time point; *T* = 0, 1, 2, 3, 4, 5, 6, 12, 16, or 24 h postgermination). Each time point is color coded.

Principal-component analysis (PCA) of TMM normalized read counts per gene (Fig. 2) showed that the biological replicates grouped closely together, with time points grouping into 3 clusters separated by time (principal component 1 [PC1]) and stage (PC2), as determined by *k*-means clustering (see Fig. S2 in the supplemental material).

10.1128/mSphere.00403-18.2 FIG S2 Sum of squares cluster analysis of PCA data. Download FIG S2, TIF file, 0.4 MB. Copyright © 2018 Sephton-Clark et al. 2018 Sephton-Clark et al. This content is distributed under the terms of the Creative Commons Attribution 4.0 International license.

### Dormant spores are transcriptionally unique.

**FIG 3:**
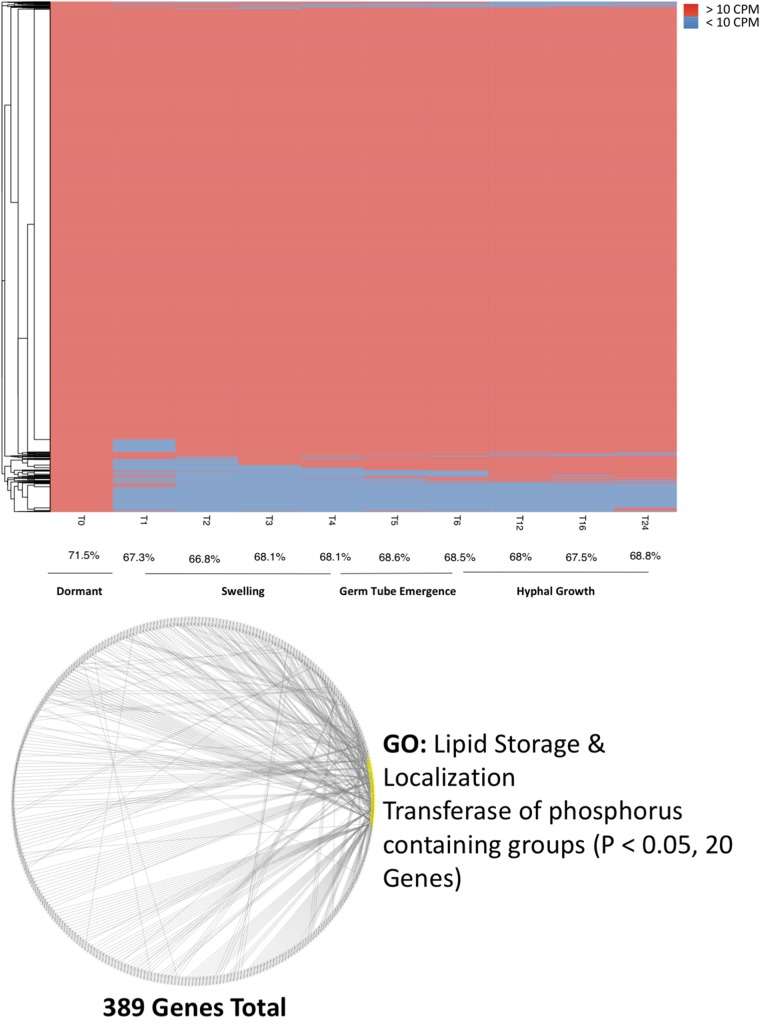
Resting-spore-specific expression. (Top) Heat map displaying the absence (blue) or presence (red) of 10 or more transcripts for a given gene over time. The average percentage of the transcriptome expressed at any given time point is given below. (Bottom) Coexpression diagram, where each node represents a gene only expressed in ungerminated spores. Nodes linked to 10 or more others are highlighted in yellow, with their functions shown adjacent.

In examining the overall transcriptional profiles of our cells, we observed a set of 482 transcripts that were only expressed in ungerminated spores (Fig. 3, top, time 0 h [T0]), representing 3.76% of total transcripts expressed in ungerminated spores (Fig. 3). As a result, genes expressed in resting spores account for 71.5% of all genes in the genome, whereas the highest percentage of the genome covered by germinated spores is 68.8% (Fig. 3, top, time 24 h [T24]). Resting-spore-specific transcripts that were coexpressed with other resting-spore-specific transcripts have predicted roles in lipid storage and localization, as well as transferase activity on phosphorous-containing compounds (Fig. 3, bottom). As these transcripts are absent in germinated spores, they may have roles in the maintenance of spore dormancy.

### Clustering of transcriptional changes over time.

**FIG 4:**
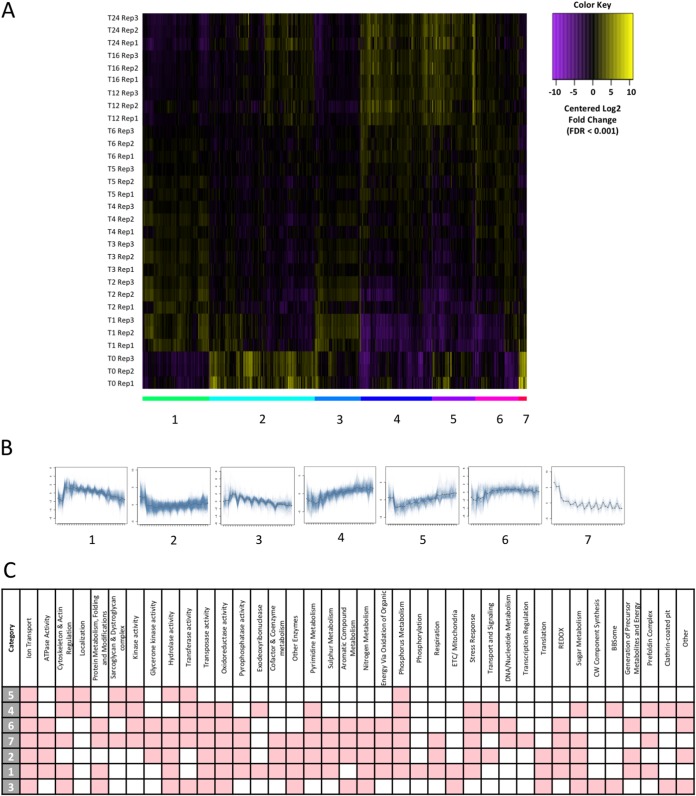
Clustering of expression over time. (A) Heat map displaying differentially expressed genes. Expression levels are plotted in log_2_, space and mean centered (FDR of <0.001) across the entire time course. *k*-means clustering has partitioned genes into 7 clusters, as indicated by colored bars and numbered graphs below the heat map. (B) Graphs displaying cluster expression over time (0 to 24 h). (C) Table displaying categories enriched (hypergeometric test, corrected *P* value of <0.05), indicated in red, for clusters 1 to 7.

We performed a series of analyses to identify the transcriptional changes occurring during spore germination (see Materials and Methods). PCA highlighted that the fungal transcriptome displayed a time-dependent shift across 3 major clusters corresponding to the phenotypic developmental stages swelling, germ tube emergence, and hyphal growth, indicating that spore germination is underpinned by progressive shifts in transcriptional regulation (Fig. 2). The transcriptome of resting spores was distinct from that of all other developmental stages, changing dramatically between 0 and 1 h. Thereafter, the transcriptional profiles of swelling spores and of those developing germ tubes were distinct but clustered together (2 to 6 h). Furthermore, fully established filamentous growth was characterized by a specific transcriptional signature (12, 16, and 24 h) (Fig. 2). Consistent with stage-specific transcriptional changes, progressive change in differential gene expression was observed during examination of the transcriptional profiles of each time point. A total of 7,924 genes were differentially expressed across the entire time course (Fig. 4A).

Analysis of differentially expressed genes by *k*-means clustering identified seven major clusters of expression variation over time (Fig. 4). Genes in clusters 1 and 3 are expressed at low levels in resting spores, with abundance increasing upon germination (1 h) (Fig. 4B). Both clusters are enriched (hypergeometric test, corrected *P* value of <0.05) for transcripts with predicted roles in regulation of the cytoskeleton, protein metabolism, the electron transport chain, translation, and sugar metabolism (Fig. 4C; see Table S1 in the supplemental material), suggesting these processes are important for germination initiation. Clusters 4 and 6 show gene expression levels moving from low to high over time, peaking during hyphal growth (Fig. 4B). These clusters are enriched (hypergeometric test, corrected *P* value of <0.05) for transcripts with predicted functions related to kinase, transferase, transposase, and oxidoreductase activities, along with pyrimidine and phosphorous metabolism, stress response, transport, and signaling (Fig. 4C; Table S1). This is consistent with the established roles for these processes in starting and maintaining vegetative growth (28, 39–41). Cluster 5 contains genes that have high expression levels in both ungerminated spores and the hyphal form, but low levels during initial swelling (Fig. 4B). Cluster 5 is enriched (hypergeometric test, corrected *P* value of <0.05) for transcripts with predicted functions in regulation of the cytoskeleton, transferase and hydrolase activities, and phosphorous metabolism (Fig. 4C; Table S1). This suggests that these functions may be repressed during isotropic growth to maintain swelling. Clusters 7 and 2 contain genes with expression levels peaking in ungerminated spores (Fig. 4B). These clusters are enriched (hypergeometric test, corrected *P* value of <0.05) for transcripts with predicted functions relating to glycerone kinase, pyrophosphatase, transferase, hydrolase, and oxidoreductase activities, as well as cofactor and coenzyme metabolism, pyrimidine, sulfur, nitrogen, sugar, and aromatic compound metabolism. These clusters are also enriched for reduction-oxidation (redox) processes, respiration, and stress responses (Fig. 4C; Table S1). Notably, every cluster is enriched for transcripts involved in ion transport regulation, specifically potassium, sodium, and hydrogen ions. This suggests tight regulation of transmembrane transport of these particular ions is important for the survival of R. delemar.

10.1128/mSphere.00403-18.4 TABLE S1 Listing of enriched terms. Download Table S1, TXT file, 0.03 MB. Copyright © 2018 Sephton-Clark et al. 2018 Sephton-Clark et al. This content is distributed under the terms of the Creative Commons Attribution 4.0 International license.

### Pairwise comparison shows transcriptional changes over time correspond to phenotypic changes during germination.

**FIG 5:**
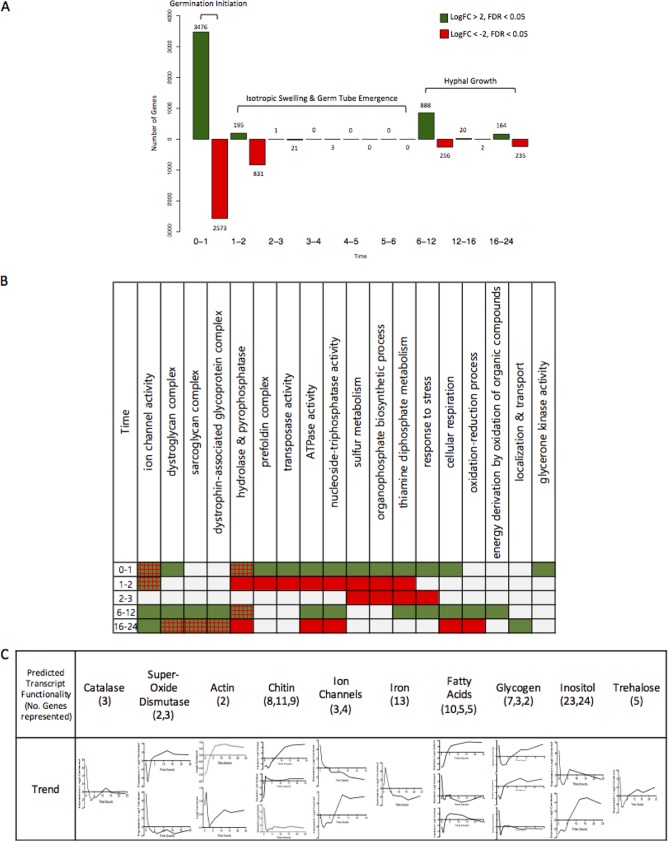
Differential gene expression over time. (A) The number of genes significantly differentially expressed (multiply corrected *P* value of <0.05) between time points, shown over time. Green bars indicate genes with an increase in expression (log fold change [FC] of >2), while red bars indicate genes with a decrease in expression (log FC of <−2). (B) Enriched categories for the up- or downregulated genes over time. Green boxes indicate an overall upregulation of this category, red indicates an overall downregulation and red-green hatching indicates mixed regulation of this category. (C) Expression profiles of transcripts in specific categories over time, with the number of transcripts represented by each trend shown in parentheses.

Ungerminated spores have a radically different expression profile from germinated spores (6,456 significantly differentially expressed genes; false-discovery rate [FDR] of <0.001): this is reflected by the functions of transcripts enriched in ungerminated spores. By pairwise comparisons of differentially expressed genes between time points, the largest transcriptional changes were seen during the first hour of germination (3,476 genes upregulated and 2,573 genes downregulated [Fig. 5A]). This was followed by a period of transcriptional consistency over the course of isotropic swelling, where few or no genes were found differentially expressed (Fig. 5A). A noticeable shift in differential expression then bridges the beginning and later stages of hyphal growth (6 to 12 h [Fig. 5A]). At the beginning of germination, an increase is observed in expression of transcripts with predicted roles in stress response, mitochondrial ribonucleases (MRP), the prefoldin complexes, organophosphate and sulfur metabolism, and transposase, ATPase, nucleoside triphosphatase, and glycerone kinase activities (Fig. 5B). A decrease in expression of genes with predicted functions in the organization of the actin cytoskeleton, carbohydrate metabolism, translation initiation factors, hexon binding, and phosphodiesterase, arylformamidase, galactosylceramidase, and precorrin-2 dehydrogenase activities is also seen (Fig. 5B). Notably, some categories are both positively and negatively regulated at the beginning of germination: transcripts predicted to have roles in ion channel activity and hydrolase and pyrophosphatase activities do not always trend together (Fig. 5B). It is likely these processes may involve several regulatory mechanisms implicated in initializing germination.

After initiation (1 to 2 h), there is an overall trend of downregulation. The majority of transcripts that were upregulated at 1 h are downregulated at 2 h (Fig. 5B), suggesting a reorganization of the transcriptome upon germination initiation. Notably, metabolism of sulfur, organophosphate, and thiamine diphosphate remains downregulated at both 2 and 3 h. After the transcriptional stability during isotropic growth and hyphal emergence, transcripts with predicted roles in stress response, respiration, ATPase and nucleoside triphosphatase activities, and redox increase during early hyphal growth (Fig. 5B). Between 6 and 12 h, the proportion of downregulated transcripts decreases, with hydrolase and pyrophosphatase activities appearing both up- and downregulated.

**FIG 6:**
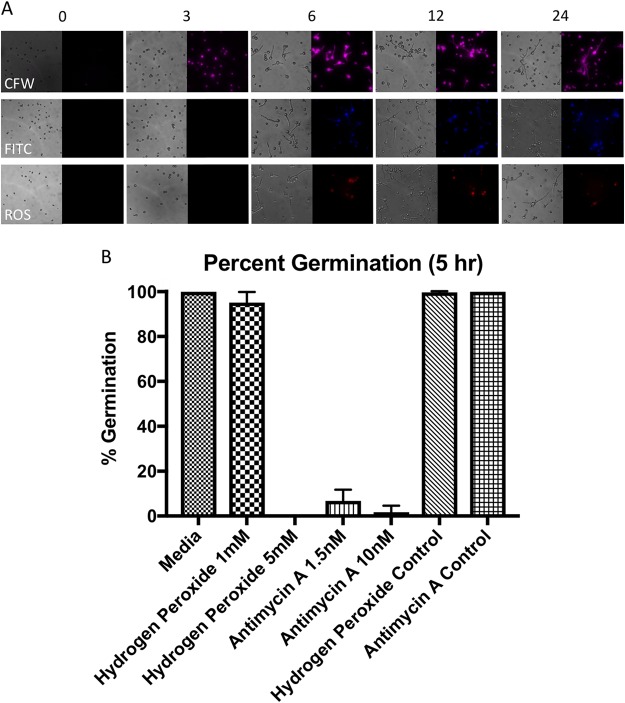
Cell wall dynamics and inhibition of germination. (A) Spores germinated for 0, 3, 6, 12, and 24 h, stained with calcofluor white (CFW), fluorescein (FITC), and ROS stain carboxy-H_2_DCFDA (ROS). (B) Germination is inhibited by 5 mM hydrogen peroxide and over 1.5 nM antimycin A, as determined by live-cell imaging, after 5 h of germination in SAB. The hydrogen peroxide control consists of an equivalent volume of H_2_O, and the antimycin A control consists of an equivalent volume of 100% ethanol.

By examining expression profiles of predicted genes with biologically interesting functions (Fig. 5C), we observe that iron acquisition transcripts rapidly increase during the initial phase of germination. This is consistent with literature that suggests iron scarcity induces abnormal germination and growth phenotypes in Mucorales species (42). Expression profiles for classes of genes related to actin, chitin, and ion channels showed two or more contrasting trends (i.e., genes with the same class do not always travel together). However, when the opposing profiles are viewed simultaneously, we see upregulation in both ungerminated spores and the hyphal form. Phenotypic data indicate that the availability of chitin (calcofluor white [CFW] stain) (Fig. 6A) within the cell wall increases rapidly over time, with spore cell walls containing high levels by 3 h. The increase in cell wall protein content, denoted by fluorescein isothiocyanate (FITC) staining (Fig. 6A), also increases over time, with high concentrations present by 6 h. Levels of transcripts involved in the production and activity of trehalose, known as a stress response molecule in fungi (43), are also high in resting spores, but decrease upon initiation of germination. Consistent with a primed stress response, we observed that the reactive oxygen species (ROS) effectors SOD (Cu/Zn and Fe/Mn superoxide dismutase) and catalase have increased expression levels in resting spores. These levels then decrease once germination is initiated, suggesting that a protective ROS stress response is involved in germination, perhaps to internal ROS produced through metabolic activity. We measured the production of endogenous ROS over time during germination (Fig. 6A). We observed that the level of endogenously generated ROS within spores increases over the course of germination, but is limited to the spore body following germ tube emergence. We investigated the significance of ROS detoxification during germination by testing for resistance to exogenous (H_2_O_2_) and endogenous (mitochondrial-derived) ROS (Fig. 6B). Treatment with 5 mM but not 1 mM H_2_O_2_ was sufficient to inhibit spore germination. In contrast, spores were highly sensitive to treatment with 1.5 or 10 nM antimycin A, a mitochondrial inhibitor that impairs cytochrome *c* reductase activity leading to the accumulation of superoxide radicals within the cell. The impact of antimycin A on germination may be 2-fold, as we also observed that the expression of storage molecule transcripts appears high in both ungerminated spores and the hyphal form. High sensitivity to inhibition of oxidative phosphorylation with antimycin A is consistent with reports that utilization of these storage molecules as energy reserves is important for the initiation and maintenance of growth (44–46).

### Transcriptional hallmarks of germination are conserved across species, while *R. delemar* exhibits unique germination responses lacking in *Aspergillus niger*.

**FIG 7:**
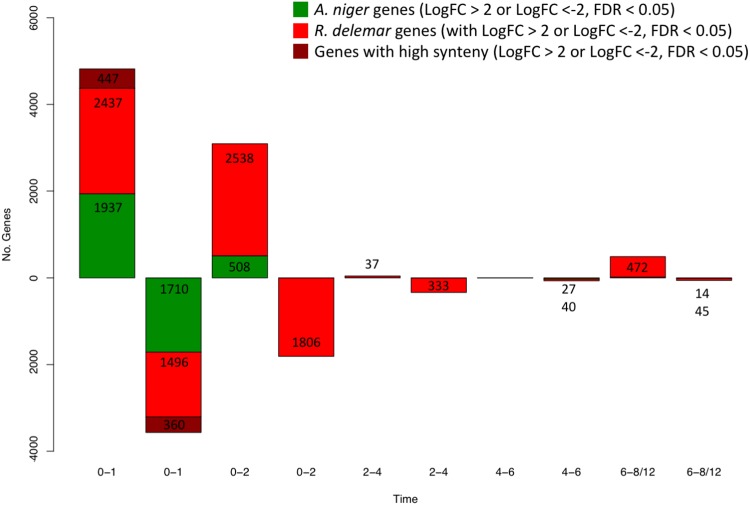
Number of homologous genes significantly differentially expressed (multiply corrected *P* value of <0.05) between time points, shown over time. Green represents the number of A. niger genes, red represents the number of R. delemar genes, and dark red represents the number of R. delemar genes found in high-synteny regions of the R. delemar genome.

It is unclear whether the mechanisms that underpin germination are conserved throughout the diverse fungal kingdom. To explore the extent of conservation, we compared our data set to other available transcriptional data sets for Aspergillus niger (see Materials and Methods). When expression profiles of homologous genes from A. niger and R. delemar are compared over the course of germination, genes with common or unique functions specific to that time point can be identified. The largest shift in the transcriptional landscape of A. niger can be seen at the initial stage of germination (26, 28); we also observed this shift in R. delemar (Fig. 7). Transcripts with predicted functions involved in transport and localization, proteolysis, and glucose, hexose, and carbohydrate metabolism increase at the initial stages of germination in both A. niger and R. delemar, while transcripts with predicted functions in translation, tRNA and rRNA processing, and amine carboxylic acid and organic acid metabolism decrease. We also observe differences between the two data sets: over isotropic and hyphal growth, homologous genes with predicted functions in valine and branched-chain amino acid metabolism were upregulated only in R. delemar, while homologous genes with predicted roles in noncoding RNA (ncRNA) metabolism, translation, amino acid activation, and ribosome biogenesis were downregulated exclusively in R. delemar. A 5% increase in genes that are uniquely up- or downregulated in R. delemar is found in high-synteny regions of the genome, compared to genes that are up- or downregulated in both R. delemar and A. niger. The duplicated nature of the R. delemar genome may allow for specific and tight regulation of the germination process, a feature unique to R. delemar.

It should be noted that A. niger and R. delemar were cultivated under conditions with different media. Aspergillus complete medium (ACM) (26), used to cultivate A. niger, and Sabouraud dextrose broth (SAB), used to cultivate R. delemar, both contain a complex mix of salts, inorganic nutrients, and organic components. Peptides are provided in SAB by mycological peptone, whereas peptides are provided by Bacto peptone in ACM. The main carbon source is the same for both ACM and SAB. Both media have a relatively low pH (ACM, pH 6.5; SAB, pH 5.6), and it is known that pH is important for regulating germination in both R. delemar (10) and A. nidulans (47). There are currently limited studies that address differences in gene expression, when germination is initiated in filamentous fungi, under different growth media. Growth characteristics of Aspergillus nidulans have been shown to vary when contents of media differ (48), while various growth cultivation methods also alter gene expression in Aspergillus oryzae (49). The effect of adding or removing specific organic and inorganic nutrients from media on the growth of filamentous fungi is also better understood (50–54). When comparing data sets or designing experiments to address these issues, the effects of using distinctly different media should be considered. This is an area that would benefit from further work aimed at exploring these effects.

## DISCUSSION

Regulation of germination in the Mucorales remains an underexamined area. Cues for germination include the availability of sufficient water, iron, a suitable carbon source, and pH (10, 42, 55), although the mechanisms remain unclear. This study aims to expand current knowledge on the molecular processes that determine germination.

### Dormant spores.

Ungerminated spores show the least exposure of chitin and protein in the cell wall, suggesting these constituents may be masked prior to germination. It is established that the ungerminated conidia of various Aspergillus species are coated by a layer of hydrophobins that confer hydrophobicity to the conidia, and these structures rearrange upon germination to reveal a more heterogeneous and hydrophilic surface (56, 57). It may be the case that similar structures coat the outside of R. delemar spores prior to germination, inhibiting the visualization of internal compounds such as chitin and protein. Transcripts involved in chitin processes, such as the predicted chitinases (Fig. 5C), appear at higher levels in ungerminated spores, a feature that can also be seen in the dormant spores of Aspergillus niger (28). This suggests that the turnover or degradation of the fungal cell wall may be an important process involved in the formation of the spore, the maintenance of dormancy, or the initial stages of germination. Pyrophosphatase, transferase, hydrolase, and oxidoreductase activities also appear to be important in ungerminated spores. The presence of pyrophosphates has been implicated in aiding pathogenicity and survival in nutrient-scarce environments for the fungal pathogen Cryptococcus neoformans (58). Interestingly, the signaling properties of pyrophosphates combined with inositol, also upregulated in ungerminated R. delemar spores, have been associated with metabolic regulation of yeast (59) and stress tolerance (60). There also appears to be a conserved requirement for sulfur in the early stages of germination across fungal species: sulfur and aromatic compound metabolism is upregulated in ungerminated spores of R. delemar, while sulfur metabolism is induced minutes after germination initiation in Phomopsis viticola. Sulfur has also been shown to be important for pathogenicity and the regulation of iron homeostasis in A. fumigatus (61). This may help explain the sharp increase in the levels of transcripts with predicted functions in iron recruitment upon the initiation of germination in R. delemar (Fig. 5C). Compared to all growth states, resting spores also show an upregulation of transcripts involved in the latter stages of iron-sulfur cluster biosynthesis (see Fig. S3 in the supplemental material).

10.1128/mSphere.00403-18.3 FIG S3 Upregulation (red) of the Fe-S cluster biosynthetic pathway, determined with PathwayTools. Download FIG S3, TIF file, 16 MB. Copyright © 2018 Sephton-Clark et al. 2018 Sephton-Clark et al. This content is distributed under the terms of the Creative Commons Attribution 4.0 International license.

Ungerminated spores are also enriched with transcripts involved in nitrogen metabolism. Nitrogen-containing compounds have been shown to trigger germination in A. niger, correlating with the upregulation of transcripts involved in nitrogen utilization during the initial stages of germination (28, 62).

Ungerminated R. delemar spores were also enriched for transcripts with roles in redox processes, respiration, and stress responses. Predicted catalase, Cu/Zn, and Fe/Mn superoxide dismutase genes appeared highly expressed in ungerminated spores, suggesting that they may form part of the stress response, as they are often utilized to resist internal metabolic ROS, as well as harsh conditions (63). An increased level of transcripts with predicted functions in the synthesis and phosphorylation of the stress response molecule trehalose (43) was also found in ungerminated spores (Fig. 4C). This suggests regulation of trehalose processes may also be implicated in the resistance to harsh conditions by R. delemar spores.

Interestingly, transcripts only present in the ungerminated spores of R. delemar had roles in lipid storage. Lipid droplets have been observed in the spores of Schizosaccharomyces pombe, where it is thought they serve as energy reserves in nutritionally poor environments (64). It is likely these transcripts play roles in maintaining lipid storage molecules, crucial for spore survival in nutritionally scarce environments. Other transcripts unique to ungerminated spores had predicted roles in transference of phosphorous groups. Transcripts involved in the degradation of the phosphorous storage molecule phytate also appeared to be upregulated in ungerminated R. delemar spores, but downregulated upon the onset of germination. This indicates spores may depend on phosphorous reserves for the initiation of germination.

### Swelling spores.

During isotropic growth, the available chitin, protein, and spore ROS contents increase, and this is reflected by changes in the transcriptome. Transcripts predicted to play roles in cell wall biogenesis, protein synthesis and protein modification are enriched in cluster 3 (Fig. 4), which shows an immediate increase in expression levels upon initiation of isotropic growth. The observation that alterations in the structure and composition of the cell wall are seemingly required for germination suggests that identification of potential methods of inhibiting germination and therefore invasive infection is possible. For example, treatment with inhibitors of chitin synthesis and transporter machinery might offer a solution for inhibiting isotropic growth and germination (65). Predicted ROS scavenger transcripts such as catalase and some SODs are also downregulated after germination is initiated (Fig. 5C). This correlates with the observation of increased levels of ROS in germinated spores. There does appear, however, to be a separate subset of Cu/Zn and Fe/Mn SOD transcripts that also remain abundant over time (Fig. 5C, upper panel), providing a possible explanation as to why the swollen and hyphal forms are able to withstand the increased levels of ROS internally. ROS and SOD activities may also be involved in directing hyphal growth (40, 66), or they may serve as signaling or metabolic molecules through compartmentalization (67).

Clusters with expression levels that increase as isotropic growth begins (Fig. 4) are also enriched in transcripts with predicted roles in the electron transport chain, translation, and sugar metabolism. This suggests that respiration is a key metabolic process utilized throughout isotropic growth. Phenotypic data showing the inhibition of germination with antimycin A also suggest this (Fig. 6B). Protein synthesis is also required to manufacture new cellular machinery and prepare for hyphal emergence. A. niger also shows an increase in the production of transcripts involved in translation and respiration upon germination of conidia (28). Again, common themes in germination involving major category classes appear conserved throughout multiple families of filamentous fungi.

After the initial transcriptional shift, a higher proportion of transcripts are then downregulated by 2 h (Fig. 5). As the downregulated transcripts are mainly those that were upregulated at 1 h, this “downregulation” may be an artifact, as transcripts potentially essential for the initiation of germination are turned over or degraded following their use. Similarly, A. niger shows a vast downregulation of transcripts between 1 and 2 h postinitiation, although whether the majority of downregulated transcripts at this time point in A. niger are found in those upregulated at 1 h has not been explored (26).

Notably, metabolism of sulfur, organophosphate, and thiamine diphosphate is downregulated for initial and mid-isotropic growth. Again, sulfur utilization in A. niger appears to be underrepresented when transcripts from conidia having germinated for 2 h are compared to those found in ungerminated conidia (28).

### Hyphal growth.

Hyphal samples were enriched for transcripts with predicted functions in kinase and oxidoreductase activities, as well as stress response and pyrimidine and phosphorous metabolism. Oxidoreductase is commonly used by the hyphal forms of wood-decaying filamentous fungi, such as Phlebia radiata and Trichaptum abietinum, thought to be useful for lignin decay (68). R. delemar is known to grow on plants with complex carbon sources (69), and the increased production of oxidoreductase may allow for the degradation of a variety of carbon sources, thus, enabling R. delemar to colonize a variety of environments.

ROS levels appear to peak in the swollen bodies of hyphal R. delemar, while levels of transcripts with predicted functions in stress response also increase. Stress response genes have been shown to be important for the hyphal growth of the filamentous fungal plant pathogens Fusarium graminearum and Ustilaginoidea virens (70, 71). Furthermore, harsh environmental conditions can induce the production of ROS internally. For example, changes in osmolarity induce hydrogen peroxide bursts within the hyphae of F. graminearum (72). This remains to be studied in Mucor species. One of the central oxidative stress response transcription factors, Yap1, is found in a range of fungi, including Candida albicans, Aspergillus fumigatus, and Neurospora crassa, and is essential for responding to ROS stress. When knocked out in Epichloë festucae, hyphae are susceptible to ROS (73). Unexpectedly a *YAP1* homologue could not be found in the genome of R. delemar. Together, our data highlight a role for ROS stress response in R. delemar germination and hyphal growth and suggest differences with other better-studied filamentous fungi.

During hyphal growth, functions enriched also included regulation of the cytoskeleton and phosphorous metabolism. The cytoskeleton is known to be important for hyphal extension, allowing the transport of vesicles to the hyphal tip to attain and maintain polarity (74, 75). Although phosphorous metabolism in the hyphae of filamentous fungi is not as well studied, it has been shown that phosphorus levels in the soil can effect germination and hyphal extension length in mycorrhizal fungi (76).

As hypothesized, transcripts with predicted roles in respiration also appear to peak around hyphal growth in R. delemar. This appears to be a conserved trait across filamentous fungi, as a higher respiratory rate is commonly seen in the hyphal form of Trichoderma lignorum (77), and increased levels of respiratory transcripts are present in hyphae of N. crassa (21).

The results of this study increase our understanding of the molecular mechanisms controlling germination in R. delemar. We have shown that ungerminated spores are transcriptionally unique, while the initiation of germination entails a huge transcriptional shift. ROS resistance and respiration are required for germination to occur, while actin, chitin, and cytoskeletal components appear to play key roles initiating isotropic swelling and hyphal growth. R. delemar shares many transcriptional traits with A. niger at germination initiation; however, transcriptional features unique to R. delemar indicate that the duplicated nature of the genome may allow for alternative regulation of this process. This study has provided a significant overview of the transcriptome of germinating spores and expanded current knowledge in the Mucorales field.

## MATERIALS AND METHODS

### Culture.

R. delemar was cultured with Sabouraud dextrose agar or broth (10 g/liter mycological peptone, 20 g/liter dextrose), sourced from Sigma-Aldrich, at room temperature. Spores were harvested with phosphate-buffered saline (PBS), centrifuged for 3 min at 3,000 rpm, and washed. Appropriate concentrations of spores were used for further experiments.

### Live-cell imaging, staining, and inhibition.

Images of 1 × 10^5^ spores/ml in SAB were taken every 10 min to determine germination characteristics. Images were taken at 20× objective on a Zeiss Axio Observer. Calcofluor white (CFW), fluorescein isothiocyanate (FITC) (Sigma-Aldrich), and the ROS stain carboxy-H_2_DCFDA (6-carboxy-2′,7′-dichlorodihydrofluorescein diacetate [C400]; Invitrogen) were incubated with live spores, according to the manufacturer’s instructions, prior to imaging. To assess inhibition, spores were incubated with 1 to 5 mM hydrogen peroxide or 1.5 to 10 nM antimycin A (Sigma-Aldrich) prior to imaging. Bright-field and fluorescent images were then analyzed using ImageJ V1.

### RNA extraction and sequencing.

Total RNA was extracted from R. delemar spores that germinated in SAB for 0, 1, 2, 3, 4, 5, 6, 12, 16, and 24 h. To extract total RNA, the washed samples were immediately immersed in TRIzol and lysed via bead beating at 6,500 rpm for 60 s. Samples were then either immediately frozen at −20°C and stored for RNA extraction or placed on ice for RNA extraction. After lysis, 0.2 ml of chloroform was added for every 1 ml of TRIzol used in the sample preparation. Samples were incubated for 3 min and then spun at 12,000 × *g* at 4°C for 15 min. To the aqueous phase, an equal volume of 100% ethanol (EtOH) was added, before the samples were loaded onto RNeasy RNA extraction columns (Qiagen). The manufacturer’s instructions were followed from this point onwards. RNA quality was checked by Agilent, with all RNA integrity number (RIN) scores above 8 (78). One microgram of total RNA was used for cDNA library preparation. Library preparation was done in accordance with the NEBNext pipeline, with library quality checked by Agilent. Samples were sequenced using the Illumina HiSeq platform; 100-bp paired-end sequencing was employed (2 × 100 bp).

### Data analysis.

FastQC (version 0.11.5) was employed to ensure the quality of all samples, a Phred value of over 36 was found for every sample. Hisat2 (version 2.0.5) was used to align reads to the indexed genome of Rhizopus delemar found on JGI (PRJNA13066) (35, 79). HTSeq (version 0.8.0) was used to quantify the output (80). Trinity and edgeR (version 3.16.5) were then used to analyze differential expression (81, 82). Pathway Tools (version 21.0) was used to obtain information on specific pathways (83). The genome of R. delemar was reannotated by incorporating the RNA-Seq data via BRAKER (version 2.1.0), this was fed into the Broad Institute annotation pipeline, which removed sequences that overlapped with repetitive elements, numbered, and named genes as previously described (84). Completeness of annotation was analyzed with BUSCO (version 3) (37, 38).

### Data availability.

Raw data and a compiled count matrix can be obtained under the following accession numbers: SRP146252 (SRA) and GSE114842 (GEO).
