# Supplementary figures and images for: Pathways of Pathogenicity: Transcriptional Stages of Germination in the Fatal Fungal Pathogen Rhizopus delemar
Source: mSphere. 2018 Sep 26;3(5):e00403-18. doi: 10.1128/mSphere.00403-18 (PMC6158513; doi:10.1128/mSphere.00403-18)

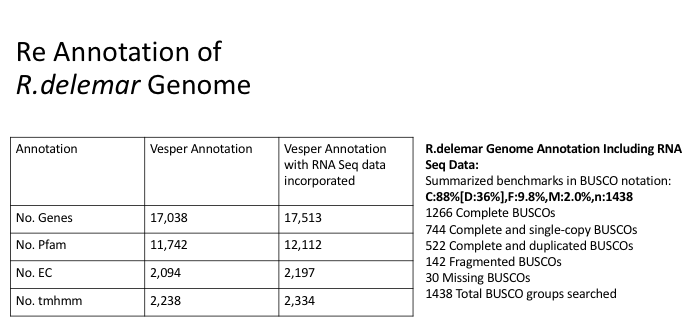

Supplement: FIG S1 [file sph005182645sf1.tif]

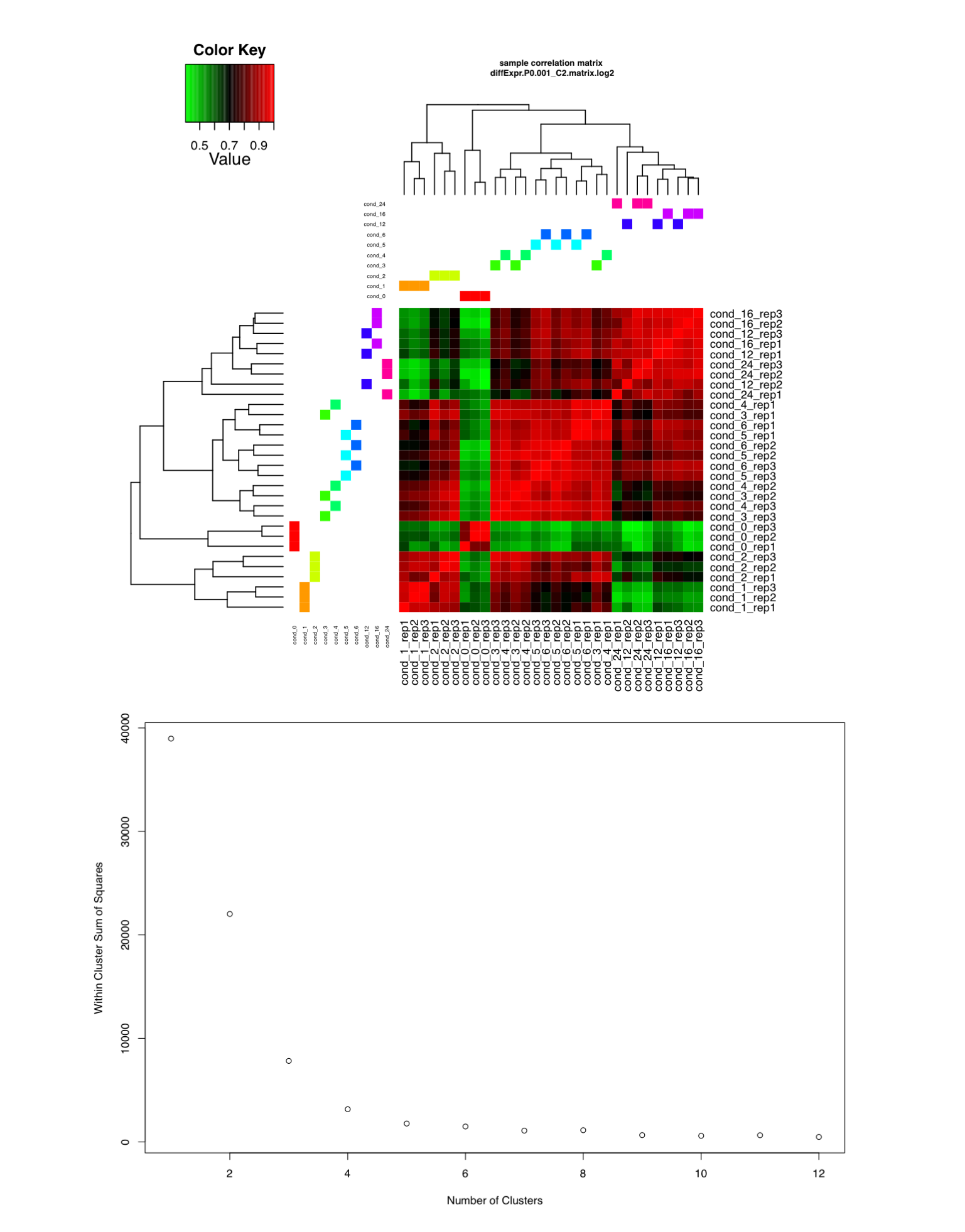

Supplement: FIG S2 [file sph005182645sf2.tif]

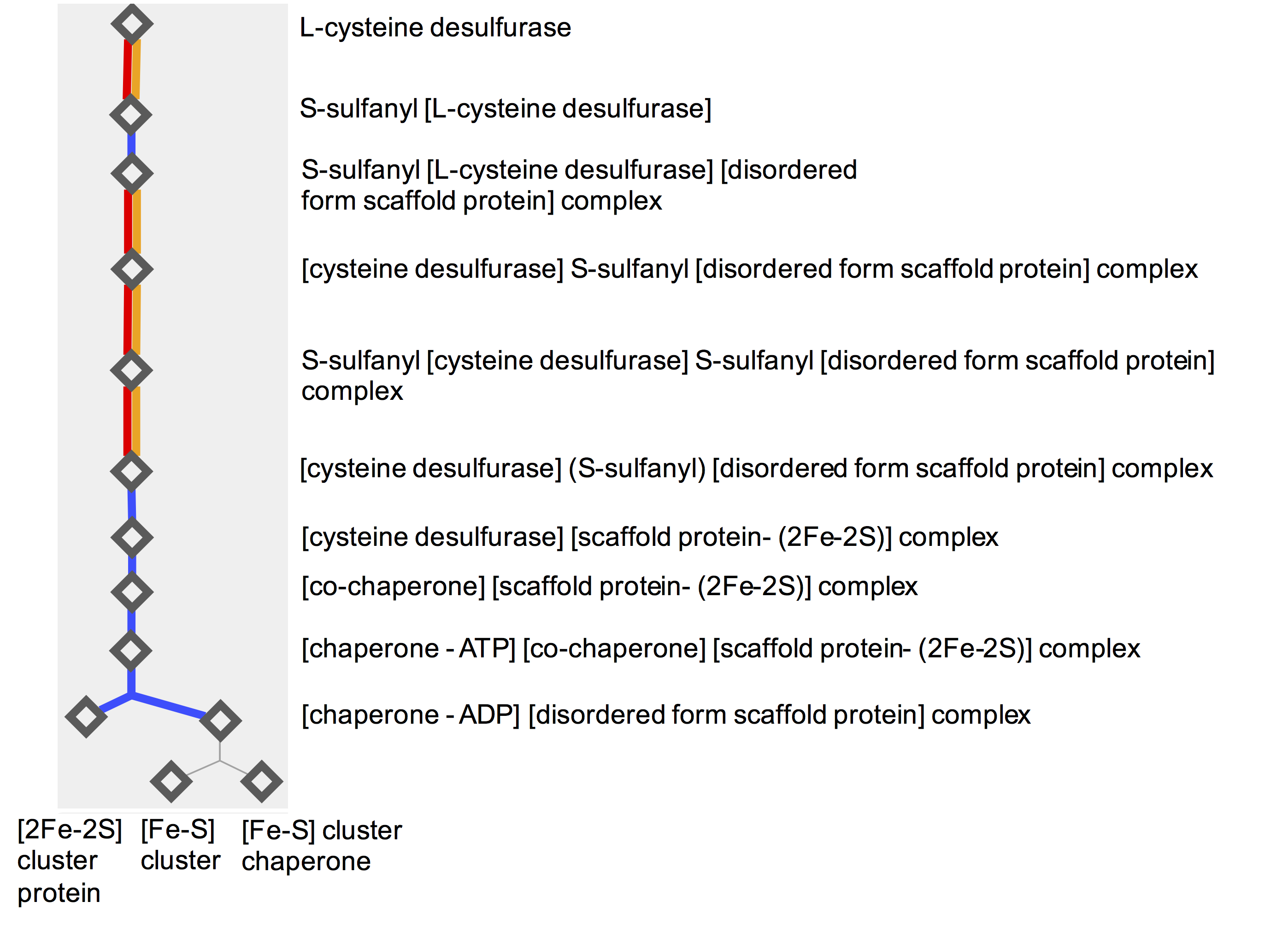

Supplement: FIG S3 [file sph005182645sf3.tif]
